# How Has the COVID-19 Pandemic Been Associated with Emergency Department Leave Without Being Seen Rates? A Comparison Between Hospitals in Ancona (Italy) and Gran Canaria (Spain)

**DOI:** 10.3390/medicina62061055

**Published:** 2026-05-29

**Authors:** Ilaria Roncarati, Laura Tomaino, Silvia Rodríguez-Mireles, Eva Rivas-Wagner, Carlo La Vecchia, Eva Negri, Valerio Di Maio, Susanna Contucci, Lorenzo Falsetti, Gianluca Moroncini, Lluìs Serra-Majem

**Affiliations:** 1 Emergency Medicine Residency Program, Marche Polytechnic University, 60126 Ancona, Italy; ilaria.roncarati@gmail.com (I.R.); laura.tomaino@gmail.com (L.T.); 2 Research Institute of Biomedical and Health Sciences, University of Las Palmas de Gran Canaria, Canary Health Service, 35016 Las Palmas de Gran Canaria, Spain; lluis.serra@ulpgc.es; 3 Department of Admissions and Clinical Documentation, Hospital Universitario de Gran Canaria Dr. Negrín, Canary Health Service, 35010 Las Palmas de Gran Canaria, Spain; srodmir@gobiernodecanarias.org; 4 Department of Epidemiology and Prevention, Public Health General Directorate, Canary Health Service, 35003 Las Palmas de Gran Canaria, Spain; erivwag@gobiernodecanarias.org; 5 Department of Clinical Sciences and Community Health, University of Milan, 20133 Milan, Italy; carlo.lavecchia@unimi.it; 6 Department of Medical and Surgical Science, University of Bologna, 40126 Bologna, Italy; eva.negri@unibo.it; 7 Department of Emergency Medicine and Surgery, Short-Term Observation, Hospital of Bolzano, 39100 Bolzano, Italy; vrmdimaio@gmail.com; 8 Emergency Unit, Marche University Hospital, 60126 Ancona, Italy; susanna.contucci@ospedaliriuniti.marche.it; 9 Department of Clinical and Molecular Sciences, Marche Polytechnic University, 60126 Ancona, Italy; 10 Clinica Medica, Department of Internal Medicine, Marche University Hospital, 60126 Ancona, Italy

**Keywords:** COVID-19, emergency department, LWBS, crowding, health centre

## Abstract

*Background and Objectives*: The COVID-19 pandemic was associated with major disruptions, not only at the environmental, social, and economic levels but also in the public health systems and, therefore, emergency care utilisation. Prior to the pandemic, one of the most significant issues in the ED was overcrowding, with a consequent percentage of people leaving the ED without being seen (LWBS). The aim of this study was to assess the association between the 2020 COVID-19 pandemic and the number of LWBS, compared with the rates recorded during the same period in 2019 and 2021. *Materials and Methods:* A retrospective comparative observational study of ED admissions was conducted in two university hospitals in Ancona (Italy) between 9 March and 3 May 2020 and in Gran Canaria (Spain) between 14 March and 10 May 2020, corresponding to the lockdown in the two countries, respectively. ED visits were assessed during the defined periods, separately for the Italian and Spanish contexts and between groups, comparing the two contexts for each year. *Results:* In Italy, during the 2019 timeframe, 597 (7.0%) of 8568 patients who arrived in the ED left before being seen; during the same period in 2020, 100 (3.2%) of 3100; and in 2021, 334 (6.0%) of 5555. In Spain, patients leaving the ED prior to medical consult in 2019 were 567 (4.0%) out of 14,034 visits; in 2020, they amounted to 185 (2.6%) out of 7208; and in 2021, they were 528 (4.0%) out of 13,214. The results of the logistic regression analysis for Italy and Spain showed that male sex [odds ratio (OR), 95% confidence interval (CI): 1.38 (1.24–1.53)], age group between 17 and 43 years compared to those subjects older than 74 years old [OR (95%CI): 4.04 (3.34–4.88)] and a lower priority code at triage were significantly associated with a higher odds of LWBS. *Conclusions:* The findings suggest that the COVID-19 pandemic was associated with changes in only some characteristics of the profiles and types of patients leaving the ED, while it had a strong impact on the number of patients who left the ED before medical examination. The observed decrease in ED visits and LWBS rates in 2020 suggests that the COVID-19 pandemic has changed the population’s use of the ED, highlighting the potential need for improved public and professional awareness of appropriate care pathways and the role of health professionals in them.

## 1. Introduction

The COVID-19 pandemic was associated with major disruptions across many aspects of our society, not only in the environmental, social, and economic fields but also in the utilisation and functioning of healthcare systems (HSs). In particular, the pandemic exacerbated the dysfunction of those HSs that have undergone progressive spending revisions and unfavourable policy choices over the past decades [1]. In particular, the work of emergency departments (EDs) changed substantially as confinement and lockdown strategies had major consequences for health service utilisation in several countries [2]. Specifically, during COVID-19, the patients’ flow through the ED was mainly due to COVID-related complaints, and dedicated pathways were implemented, thus affecting the management of all the other conditions not considered as emergencies. Despite similar organisation of the ED and comparable accessibility pathways to the Emergency Service for the population, Italy and Spain differ in primary care services. In Italy, territorial healthcare is mainly composed of general practitioners, professionals who provide primary care, out-of-hospital medical specialists, and residential assistance for the population [3]. These services are maintained during working days and daytime hours; during nocturnal hours and holidays, the territorial medicine service is guaranteed by “continuity of care” services. In Spain, primary care is provided by health centres, which are widely distributed across the territory, offering all individuals diagnostic, therapeutic, and follow-up care for acute or chronic conditions, as well as health promotion, health education, and disease prevention activities carried out by various primary care professionals. Prior to the pandemic, one of the most critical issues in the ED was overcrowding, resulting in long waiting times before medical examination for low-priority patients. This phenomenon led to self-discharge, commonly known in the literature as “people who left without being seen” (LWBS) by physicians [4]. The number of patients who LWBS seems to vary between hospitals and countries, ranging from 0.84% to 15% in the United States, 1.1% to 10.1% in Australia, with an estimated average of 3.26% in England. The lowest rates were observed in Asia: 0.36% in Hong Kong and 0.1% in Taiwan [5,6]. Only a limited number of studies are available on the Italian ED system. In 2011, a study by the Azienda Ospedaliero-Universitaria Careggi in Florence aimed to describe the demographic and clinical characteristics of patients discharged from the ED and of those who left the ED before the medical examination [7] and showed very low levels of LWBS (2%). In 2015, the average ED rate of LWBS in Italy was 2.1%, ranging between 0.1% (Friuli Venezia Giulia Region) and 5.9% (Lazio Region) [8]. Moreover, in Spain, there are limited data available on this phenomenon. In 2007, a study conducted at the “Puerta de Hierro” University Hospital in Madrid showed 0.8% of LBWS [9]. In 2016, the University Hospital of Lleida showed an LWBS rate of 3.5% [10]. The lack of uniformity in ED abandonment rates must be attributed to multiple factors, including differences in the definition of the LWBS category itself, as well as cultural differences and local policies [5].

Therefore, considering the similarities in the ED organisation and the differences in the primary healthcare provision, this study aims to assess the association between the COVID-19 pandemic and the number of patients who left the ED without being seen and those who left during diagnostic investigations in 2020 and to compare these rates with those recorded during the same period in 2019 and 2021 at two university hospitals in Italy and Spain. Differences in primary care accessibility and organisation may influence non-urgent ED utilisation and consequently LWBS rates.

## 2. Materials and Methods

We conducted a retrospective observational comparative study including the rate of subjects who left the ED prior to medical discharge among all the ED accesses to two university hospitals: “Azienda Ospedaliero Universitaria delle Marche” (Ancona, Italy) and the “Hospital Universitario de Gran Canaria Dr Negrín” (Gran Canaria, Spain). The two hospitals serve a resident population estimated at between 250,000 and 350,000 inhabitants. Respectively, the ED visits from 9 March to 3 May and from 14 March to 10 May 2020, corresponding to the Italian and Spanish lockdowns, were recorded and then compared with the number of admissions during the same periods in 2019 and 2021. Different lockdown dates reflected national policy timelines, but equivalent lockdown phases were compared across countries.

For the present study, the LWBS category was defined as those subjects who were triaged and registered in one of the two selected hospitals but subsequently left without any discharge by the handling physician, regardless of the progress of the diagnostic–therapeutic process.

Children under 16 years of age and pregnant women were excluded from the study as they were referred to a dedicated ED in both hospitals. For both the Italian and Spanish settings, data regarding the number of ED visits during the abovementioned timeframes were anonymously extracted, and the following variables were recorded: socio-demographic data; date, time, and mode of arrival (through emergency medical service or self-decision); priority level on arrival; a colour scale made of 4 levels, augmented to 5 from 1 February 2021 [11]; and discharge information such as outcome (discharged, hospitalised, LWBS transferred or deceased) and discharge diagnosis according to the International Classification of Diseases (ICD).

For the Italian setting, priority level at triage upon arrival was established according to the Italian guidelines, based initially on a 4-level colour scale, augmented to 5 from 1 February 2021 [11], while the diagnosis at completed ED care was classified according to the ICD—9th Revision, Clinical Modifications (ICD-9-CM) [12]. For the Spanish setting, priority level at triage was assessed according to the Emergency Severity Index (ESI) [13], a scale of ascending numbers, and the diagnosis at completed ED care was classified according to the ICD—10th Revision (ICD-10-CM) [14]. To ensure comparability between the Italian and Spanish settings, triage systems were harmonised into a unified 5-level scale, aligning the Italian colour-coded system and the ESI based on clinical urgency. This mapping was performed according to previously validated equivalence frameworks reported in the literature. Therefore, in this study, to make the levels of presentation to triage homogeneous, considering that the colours of the Italian triage system and the numbers of the ESI correspond to the same priority levels (number 1 aligns with colour red, number 2 with orange, number 3 with blue, number 4 with green, and number 5 with colour white) and waiting times, for this study, a unified classification system was adopted: ascending numbers from 1 to 5 defining descending severity. This method was previously described elsewhere by our investigation group [15]. Similarly, the diagnostic categories derived from ICD-9-CM and ICD-10-CM classifications were reorganised in disease groups (e.g., cardiovascular, respiratory, trauma/intoxication, and mental health disorders) based on organ-system classification, as described elsewhere.

Waiting time before self-discharge (or LWBS time), defined as the autonomous decision of the subject to leave the ED prior to medical discharge and regardless of the progression of the diagnostic–therapeutic process, was calculated as the date/time of abandonment prior to clinical examination minus the date/time of admission. According to the time of admission at the ED, the study sample was divided into three groups (morning, from 7 a.m. to 2 p.m.; afternoon, from 2 p.m. to 9 p.m.; and night, from 9 p.m. to 7 a.m.) corresponding to the three work shifts of the medical staff in both the study settings. All the timestamps were electronically recorded.

### 2.1. Statistical Analysis

We used the Kolmogorov–Smirnov and Shapiro–Wilk tests to assess the normality of continuous variables. If normally distributed, continuous data are presented as mean ± standard deviation (SD). Categorical variables (sex, triage priority level, mode of arrival, and disease group) are expressed as frequencies (n) and percentages (%). Participants were divided into two groups: those LWBS and those who were discharged from the ED by the handling physician after completing the diagnostic–therapeutic process, regardless of the outcome (hospitalised, transferred to other healthcare facilities, or deceased). Subgroup analyses were performed by year (2019, 2020, and 2021) and study setting (Italian and Spanish). Categorical variables were compared using Pearson’s chi-square test, while continuous variables were analysed using the analysis of variance (ANOVA) test. Multivariable logistic regression analysis was performed on the pooled dataset to identify potential determinants of LWBS, including both countries and all study years. The dependent variable was LWBS (yes/no). Independent variables entered into the multivariable logistic regression model included sex, age group, triage priority level, admission shift, study year, and study setting/hospital. Adjusted odds ratios (aOR) and corresponding 95% confidence intervals (CI) were calculated. The selection of covariates was based on prior literature identifying age, sex, triage priority, and time of presentation as key predictors of ED abandonment. In this model, the dependent variable was represented by the dichotomous variable LWBS (yes/no) to obtain the odds ratio (OR) and the corresponding 95% confidence interval (CI), while the independent variables or predictors were age (classified in groups: 17–43 years old, 43–60, 60–75, and >75 years old), priority level at triage (levels 1–2 versus 3–5), and hour of registration at triage (morning, i.e., from 7 a.m. to 2 p.m., afternoon from 2 p.m. to 9 p.m., and night from 9 p.m. to 7 a.m.). For this analysis, age was divided into quartiles (cut points: 43, 60, and 75 years), and triage priority level was dichotomised (high priority: codes 1 and 2; low priority: codes 3, 4, and 5). The analyses were carried out using the Statistical Package for the Social Sciences (SPSS^®^), version 26 (IBM Corp.), SPSS software, version 26.0 (IBM^®^ Corp.).

### 2.2. Ethical Considerations

The study was conducted in accordance with the Declaration of Helsinki and under the terms of local regulations in force. The study protocol was approved by the ethical committees of both the Italian and the Spanish centres. For the Italian setting, this study, as part of a larger analysis, was approved by the Marche Region Ethics Committee (CET Marche, year: 2022; record n. 2022/35). For the Spanish data, the study project was approved by the Spanish Ethics Committee of the Hospital Universitario de Gran Canaria Dr. Negrín, Spain (year: 2022; record n. 2022-439-1). Data were extracted anonymously and aggregated; therefore, written informed consent was not required from study participants, and the informed consent request was waived by both ethical committees that reviewed and approved the protocol before the analysis.

## 3. Results

Overall, ED visits and LWBS rates showed a marked reduction during the 2020 lockdown period in both settings, followed by a partial return to pre-pandemic levels in 2021.

Compared with the corresponding timeframe in 2019, during the 2020 lockdown period, ED visits decreased by 63.8% in Ancona (3100 versus 8568 of the previous year) and 48.6% in Gran Canaria (7208 versus 14,034, respectively) during the 2020 lockdown period. Similarly, across the two timeframes, LWBS rates decreased from 7.0% of the ED visits (n = 597) to 3.2% (n = 100) in Ancona and from 4.0% (n = 567) to 2.6% (n = 185) in Gran Canaria.

Table 1 shows the demographic characteristics of patients who accessed the Italian ED and either LWBS or remained till medial discharge from the ED, respectively, during the considered timeframes. In Ancona, in 2019, 7.0% (n = 597) of subjects who were registered in the ED left before being seen by a physician or during the diagnostic–therapeutic process, as previously described in the Section 2. This rate decreased to 3.2% (n = 100) in 2020, then increased again in 2021, when the LWBS rate reached 6.0% (n = 334) among those who registered at the ED. Among patients with LWBS, the majority were men across all three study periods: 58.1% (n = 347) in 2019, 57.6% (n = 57) in 2020, and 59.3% (n = 198) in 2021. Regarding patient age, LWBS patients were significantly younger than those who completed the hospital procedure until discharge by the handling physician, regardless of the outcome (e.g., discharged, transferred, admitted to an in-hospital facility).

As shown in Table 2, the number of patients who left before or during medical investigations in Gran Canaria was lower overall than in the Italian setting. In 2019, they represented 4.0% of those who registered at triage. In 2020, they reduced to 2.6%, then rose again in 2021, reaching 4.0% of patients who accessed the ED. Among those who LWBS in Gran Canaria, the majority were men across all the timeframes considered: 56.1% in 2019, 63.2% in 2020, and 63.4% in 2021. Like the findings regarding the Italian setting, those who had LWBS were significantly younger than those who had completed the hospital procedure.

Table 3 compares the characteristics of those who LWBS in Ancona and Gran Canaria. The time spent in the ED before leaving ranged from 4.6 h (in 2019) to 3.3 (in 2020) and 5.0 h (in 2021) in the Italian setting, while in the Spanish setting, it ranged from 4.1 h (2019) to 3.6 (2020) and 3.9 h (in 2021), with no significant differences across the two study setting during the selected timeframes.

Considering the arrival mode of those who LWBS in 2019, access by self-decision accounted for 80.1% of all ED visits in Ancona and 74.6% in Gran Canaria. During the 2020 lockdown, almost half of ED visits were made via emergency medical system (EMS). These figures, which were raised again in 2021, were 77.8% and 65.0% of patients who went to the EDs in Ancona and Gran Canaria, respectively, who arrived on their own.

According to the triage registration time, ED visits were classified into 3 groups: morning (7 a.m. to 2 p.m.), afternoon (2 p.m. to 9 p.m.), and night (9 p.m. to 7 a.m.). Across all study periods and in both the Italian and Spanish settings, in the afternoon, there were more visits by patients with LWBS than in the morning or at night. Among those who LWBS, in 2019, 42.7% in Ancona and 49.1% in Gran Canaria accessed the ED during the afternoon shift. Similar figures were observed in 2020 (44.0% and 45.7% of those who LWBS in Ancona and Gran Canaria, respectively) and in 2021 (45.5% and 46.2%, respectively).

The majority of those who LWBS accessed the hospital ED on Monday, both in the Italian and Spanish setting and over the three study periods. In Ancona, they amounted to 18.9%, 25%, and 18.9% in 2019, 2020, and 2021, respectively, while in Gran Canaria, these figures reached 19.4%, 17.3%, and 17.8% in the three study periods.

Of interest, most patients leaving the ED before discharge were assigned low priority levels at triage, e.g., 3 or 4. In Ancona, the majority of patients who left without being seen presented a priority classified as “green” (corresponding to levels 3 and 4 until February 2021 and to level 4 from February 2021 onwards) in all periods considered: 70.9% in 2019, 70% in 2020, and 62.9% in 2021. Similarly, in Gran Canaria, most patients who LWBS were assigned priority levels 3 and 4: 87.8% in 2019, 75.7% in 2020, and 83.2% in 2021.

Regarding the disease group, in the Spanish setting, the main reason for access in those who left was categorised as “Other”, as shown in Table 3. This category, which encompasses undefined signs and symptoms, abnormal test results for which patients sought medical advice, or other conditions not classified elsewhere, represented 99.3% of diagnoses among those who LWBS in 2019, 100% in 2020, and 99.6% in 2021. In Ancona, on the other hand, the reasons for access were more varied, although the majority were related to the “Other” category, trauma and/or intoxications, and mental health. This observation may be due to the fact that if a patient chooses to leave the ED before being medically discharged, regardless of how the diagnostic and therapeutic process has progressed, the treating physician archives the clinical record. When the diagnostic–therapeutic progression allows for defining a diagnostic category, this can be inserted in the patient’s record by the physician, but this procedure is not standardised and varies across hospitals and healthcare personnel themselves.

The results of the multivariable logistic regression analysis, adjusted for study year and study setting/hospital, showed that male sex was positively associated with LWBS, with an OR (95% CI) of 1.38 (1.24–1.53; *p* < 0.001). Moreover, if compared to patients aged 75 years old or older (set as the reference category), younger ages were positively associated with markedly increased odds of LWBS (OR 4.04; 95%CI: 3.34–4.88; *p* < 0.001) for those aged 17 to 43 years, moderately increased odds of LWBS (OR 3.04; 95%CI: 2.51–3.68; *p* < 0.001) for patients of 43 to 60 years old, and slightly increased odds of LWBS (OR: 1.68; 95%CI: 1.37–2.07; *p* < 0.001) for those between 60 and 75. On the other hand, compared with triage levels 3 to 5, high-priority patients (levels 1–2) showed lower odds of LWBS (OR 0.71 (0.61–0.82); *p* < 0.001). Compared to overnight hours, ED access in the morning was negatively associated with LWBS (OR 0.84; 95%CI:0.72–0.96; *p* = 0.013), while those patients who were admitted between 2 p.m. and 9 p.m. were more likely to leave before completion of ED care (OR 1.23; 95%CI:1.08–1.41; *p* = 0.002). Key determinants of LWBS across both settings included younger age, male sex, and lower triage priority, with consistent patterns observed across study periods. The results of the multivariable logistic regression analysis for the determinants of LWBS are summarised in Table 4.

The multivariable logistic regression model included sex, age group, triage priority level, admission shift, study year, and study setting/hospital as covariates. Reference categories were female sex, age > 75 years, low triage priority (levels 3–5), night admission, study year 2019, and Spain setting. Abbreviations: aOR, adjusted odds ratio; CI, confidence interval; LWBS, leave without being seen.

## 4. Discussion

The present study compared the LWBS rates in the EDs of two European settings: Ancona (Italy) and Gran Canaria (Spain). Our findings suggest that the ED of Gran Canaria had lower percentages of LWBS than the Italian one across all three timeframes (4.0% versus 7.0% in 2019, 2.6% versus 3.2% in 2020, and 4.0 versus 6.0% in 2021). Although these differences were not statistically significant, the decrease observed in 2020 was greater in Italy than Spain. A possible explanation for this difference could be that the pandemic had a more significant impact in Italy than in the Canary Islands due to differences in the speed of virus spread and in the rapid adoption of confinement measures following the Italian example. Furthermore, the Spanish primary care system is organised around health centres, which provide 24/7 medical assistance with a small ED dedicated to minor emergencies, corresponding to triage levels 3 to 5. Therefore, users with non-emergency conditions are more likely to be attended to in those structures than to wait long in the ED, as discussed elsewhere [15].

Considering the mode of arrival, in 2020, we found no significant difference between the two settings, and almost half of the patients reached the ED by EMS, compared with 2019 and 2021, when the majority arrived on their own. This may be explained on one hand by the confinement measures in place, which restrict free circulation, except for justified work and health needs, and on the other by the worsening of clinical conditions of those subjects who alerted the EMS and were therefore telephonically assessed by healthcare personnel, who, in turn, decided whether an assisted transport was needed or not. In this sense, the observed LWBS reduction, together with an increased number of ED access through the EMS, could suggest that subjects were referred to the ED for medium-to-serious health complaints, rather than light or non-urgent ones.

The harmonisation of different triage systems and diagnostic classifications represents a potential limitation. Although a standardised 5-level triage scale and grouped diagnostic categories were adopted to improve comparability, residual misclassification cannot be entirely excluded due to inherent differences between systems (ICD-9-CM versus ICD-10-CM and national triage protocols). The change in the triage system from 4 to 5 codes in Italy in 2021 has allowed for a focus on clinical conditions that fall within the field of deferrable urgency, also identifying the field of pathologies to be defined as minor urgency. The findings of this study show that the percentage of levels 3 and 4 considered together in 2021 is similar to that of level 4 in 2019 and 2020.

The findings of this study show that most patients who LWBS were assigned the diagnosis classified as “Other” or “unknown causes of morbidity and mortality”. Based on the level of progress in medical investigations before the patient’s decision to abandon the ED and before discharge, it is possible to assign a detailed diagnosis. That is true for those patients who LWBS, while a slightly more detailed discharge diagnosis is possible for those patients who leave after a specialist’s advice or a diagnostic test. Moreover, the difference observed between Ancona and Gran Canaria in this regard could be due to the presence of specialists in the health centres who handle minor emergencies in certain areas; therefore, the typology of LWBS differs from the Italian one and falls almost entirely under the heading “Other”.

Our findings are in line with the available literature on this issue, which describes the profile of patients leaving the ED without being seen by a physician as young, predominantly male, arriving on their own, with a low triage priority level and consequently longer waiting times [5]. Moreover, the findings of this study show highest LWBS rates on Monday, compared to the other weekdays. This can be explained by the fact that on Monday, general practices in Italy and health centres in Spain are particularly busy. For this reason, a large number of subjects may decide to refer directly—and in most cases, inappropriately—to the ED even for minor clinical conditions. Considering LWBS rates in our study, except for 2020, we observed higher figures than the data reported in the literature for both Italy and Spain [4,10]. Furthermore, regarding ED admission time, an Italian study found that patients arriving at night and on weekends had a higher odd of LWBS [4], but these findings were not reflected in ours.

This study suggests that the COVID-19 pandemic was associated with changes in the profile and type of patients leaving the ED with completed ED care in only a few aspects and had a large impact on the number of patients accessing the EDs of the selected hospitals. In Ancona and Gran Canaria, during the first phase of the 2020 pandemic, ED visits also decreased by more than 50% due to the lockdown. In addition, there was a drastic reduction in triage levels 5, 4, and 3, as well as in levels 2 and 1 [16].

Our results confirm a decrease in both admissions and the LWBS rate in the ED compared with the period prior to COVID-19 [17] and with 2021. However, our results are not in line with those reported by Yakobi et al., who found a decrease in ED visits during the pandemic but an increase in the LWBS rate [18]. This difference, also observed in another Italian study, could be explained by differences in demographics, culture, and local health policies that influence the LWBS rate without affecting the ED management and functioning [19].

The reduction in ED visits and LWBS rates observed in 2020 suggests that the COVID-19 pandemic significantly affected population utilisation of ED services. Those patients who used the ED as a direct gateway to care in 2019 and 2021, during 2020, due to the restrictive measures adopted, to growing concerns about the risk of infection in hospitals and media pressure for informed ED use, chose different care pathways or avoided seeking healthcare altogether. Apart from the pandemic itself, growing evidence describes different strategies that turned out to be effective in reducing ED LWBS rates, e.g., improved communication between healthcare staff and patients in the waiting room regarding the organisation of the ED and the triage system, improved waiting room layout, the presence of a physician at triage, and improved fast-tracking [5,20]. In addition, a British study found that patients after an ED episode are more likely to consult general practitioners and other community health services [5].

These considerations further reinforce the relationship between the emergency system and the primary care service and particularly the ED LWBS and the need to educate the population on appropriate care pathways, thus preferring, when presenting for chronic or not-acute complaints, territorial structures, such as the “Health Centres”, a well-established territorial reality in Spain that has also been planned to be implemented in Italy within the “National Recovery and Resilience Plan” (PNRR) framework for establishing health centres that provide the resident community with direct access to primary healthcare and social care systems.

### Limitations

This study has several limitations. First, its retrospective design may introduce selection and information biases. Second, the use of administrative data limits the granularity of clinical information and may be subject to coding inaccuracies. As many of those who registered to the ED left prior to the end of the diagnostic–therapeutic process, without the possibility to receive a detailed discharge diagnosis, most of the cases were given the “Other” diagnostic classification. Third, despite adjustment for key variables, residual confounding cannot be excluded because factors such as socioeconomic status, perceived waiting time, and organisational differences were not available. Finally, the inclusion of only two centres may limit the generalisability of the findings to other HSs.

## 5. Conclusions

The present study was conducted to compare LWBS rates in the ED during the COVID-19 lockdown in two European settings and across similar timeframes in the year preceding and following the onset of the pandemic.

The observed reduction in LWBS rates during the lockdown compared to the corresponding timeframes in 2019 and 2021 reflect changes in ED utilisation patterns during and after the pandemic. To assess whether this change is due to the differences in the primary healthcare organisation, further investigation in this field is required. Nevertheless, our findings suggest that many ED visits in the selected study settings are due to deferrable, non-urgent conditions. These data represent a starting point for considering appropriate measures at the primary healthcare level to reduce pressure on the ED.

## Figures and Tables

**Table 1:** Description of patients who left the ED and those who remained (Italy).

Ancona (Italy)									
	2019			2020			2021		
Admission to the ED	Total n = 8568	LWBS n = 597	REMAINED n = 7971	Total n = 3100	LWBS n = 100	REMAINED n = 3000	Total n = 5555	LWBS n = 334	REMAINED n = 5221
**Age, years**	*p* = 0.045			*p* = 0.056			*p* = 0.048		
	54.5 (20.2)	46.5 (17.9)	55.2 (20.2)	57.9 (19.0)	46.4 (17.4)	58.3 (19.0)	56.2 (19.8)	44.9 (17.2)	56.9 (19.8)
**Sex:** **Male** **Female**	*p* = 0.06			*p* < 0.05			*p* = 0.06		
	4.657 (54.4) 3.911 (45.6)	347 (58.1) 250 (41.9)	4.310 (54.1) 3.661 (45.9)	1.790 (57.8) 1.310 (42.2)	57 (57.6) 43 (42.4)	1.733 (57.8) 1.267 (42.2)	3.201 (57.6) 2.354 (42.4)	198 (59.3) 136 (40.7)	3.003 (57.5) 2.218 (42.5)

Continuous variables are expressed as mean (SD), categorical variables as frequency (%); *p*-values refer to comparisons between LWBS and Remained; significance set at *p* < 0.05.

**Table 2:** Description of patients who left the ED and those who remained (Spain).

Gran Canaria (Spain)									
	2019			2020			2021		
Admission to the ED	Total n = 14,034	LWBS n = 567	REMAINED n = 13,467	Total n = 7208	LWBS n = 185	REMAINED n = 7023	Total n = 13,214	LWBS n = 528	REMAINED n = 12,686
**Age, years**	*p* = 0.048			*p* = 0.035			*p* = 0.048		
	54.5 (20.2)	49.9 (18.3)	59.9 (20.4)	57.9 (19.0)	49.3 (16.3)	61.0 (19.8)	56.2 (19.8)	48.9 (17.7)	59.7 (20.4)
**Sex:** **Male** **Female**	*p* < 0.05			*p* = 0.048			*p* < 0.05		
	6.979 (49.7) 7.055 (50.3)	318 (56.1) 249 (43.9)	6.661 (49.5) 6.806 (50.5)	3.844 (53.3) 3.364 (46.7)	117 (63.2) 68 (36.8)	3.727 (53.1) 3.296 (46.9)	6.706 (50.7) 6.508 (49.3)	335 (63.4) 193 (36.6)	6.371 (50.2) 6.315 (49.8)

Continuous variables are expressed as mean (SD), categorical variables as frequency (%); *p*-values refer to comparisons between LWBS and Remained; significance set at *p* < 0.05.

**Table 3:** Characteristics of ED visits in patients who LWBS in Ancona and Gran Canaria.

	2019		2020		2021	
	Ancona	Gran Canaria	Ancona	Gran Canaria	Ancona	Gran Canaria
**Time in ED, minutes**	*p* = 0.23		*p* = 0.15		*p* = 0.16	
	278.8 (271.4)	247.3 (245.6)	199.7 (223.8)	215.0 (223.6)	302.2 (233.3)	231.0 (253.7)
**Arrival mode:** **Emergency service** **Self-decision**	*p* < 0.05		*p* = 0.075		*p* < 0.01	
	119 (19.9) 478 (80.1)	144 (25.4) 423 (74.6)	51 (51.0) 49 (49.0)	77 (41.6) 108 (58.4)	74 (22.2) 260 (77.8)	185 (35.0) 343 (65.0)
**By access shift:** **Morning (7 a.m.–2 p.m.)** **Afternoon (2 p.m.–9 p.m.)** **Night (9 p.m.–7 a.m.)**	*p *= 0.90		*p *= 0.60		*p *= 0.80	
	205 (34.3) 255 (42.7) 137 (22.9)	163 (28.8) 278 (49.1) 125 (22.1)	23 (23.0) 44 (44.0) 33 (33.0)	54 (29.3) 84 (45.7) 46 (25.0)	124 (37.1) 152 (45.5) 58 (17.4)	170 (32.2) 244 (46.2) 114 (21.6)
**By weekday:** Monday Tuesday Wednesday Thursday Friday Saturday Sunday	*p* < 0.05		*p *= 0.20		*p *= 0.12	
	113 (18.9) 81 (13.6) 70 (11.7) 73 (12.2) 102 (17.1) 91 (15.2) 67 (11.2)	110 (19.4) 69 (12.2) 81 (14.3) 93 (16.4) 104 (18.3) 54 (9.5) 56 (9.9)	25 (25.0) 16 (16.0) 17 (17.0) 8 (8.0) 15 (15.0) 10 (10.0) 9 (9.0)	32 (17.3) 29 (15.7) 32 (17.3) 24 (13.0) 25 (13.5) 21 (11.4) 22 (11.9)	63 (18.9) 60 (18.0) 50 (15.0) 47 (14.1) 35 (10.5) 45 (13.5) 34 (10.2)	94 (17.8) 83 (15.7) 89 (16.9) 74 (14.0) 93 (17.6) 43 (8.1) 52 (9.8)
**Priority level:** 1 (red) 2 (orange) 3 (blue) 4 (green) 5 (white)	*p *= 0.048		*p *= 0.037		*p *= 0.044	
	2 (0.3) 47 (7.9) - 423 (70.9) 125 (20.9)	- 54 (9.5) 261 (46) 237 (41.8) 15 (2.6)	1 (1.0) 23 (23.0) - 70 (70.0) 6 (3.2)	2 (1.1) 37 (20.0) 83 (44.9) 57 (30.8) 6 (3.2)	2 (0.6) 12 (3.6) 58 (17.4) 210 (62.9) 52 (15.6)	- 85 (16.1) 230 (43.6) 209 (39.6) 4 (0.8)
**By disease group:** Infectious Onco/haematological Endocrine/metabolic Mental health CNS disease ENT CV disease Respiratory disease Digestive Skin Musculoskeletal Genitourinary Other Trauma/intoxication	*p *= 0.002		*p *= 0.001		*p *= 0.002	
	5 (1.6) 4 (1.2) - 14 (4.3) 1 (0.3) 9 (2.8) 5 (1.6) 2 (0.6) 11 (3.4) 3 (0.9) 5 (1.6) 4 (1.2) 239 (74.2) 20 (6.2)	- - - - - 2 (0.4) 1 (0.2) - - - - - 563 (99.3) 1 (0.2)	- - - 6 (10.2) 1 (1.7) 1 (1.7) 1 (1.7) 2 (3.4) 1 (1.7) - 1 (1.7) 1 (1.7) 40 (67.8) 5 (8.5)	- - - - - - - - - - - - 185 (100) -	1 (0.9) - 1 (0.9) 7 (6.1) - 3 (2.6) 1 (0.9) 3 (2.6) 1 (0.9) 1 (0.9) 2 (1.7) 3 (2.6) 76 (66.1) 16 (13.9)	- - - 1 (0.2) - - - - - - - - 526 (99.6) 1 (0.2)

Continuous variables are expressed as mean (SD), categorical variables as frequency (%); Abbreviations: CNS: central nervous system; CV: cardiovascular; ED: emergency department; ENT: ear–nose–throat. *p*-values refer to comparisons between countries; significance set at *p* < 0.05.

**Table 4:** Multivariable logistic regression analysis for determinants of LWBS.

Variable	Reference Category	Adjusted OR (aOR)	95% CI	*p*-Value
Male sex	Female	1.38	1.24–1.53	<0.001
Age 17–43 years	>75 years	4.04	3.34–4.88	<0.001
Age 43–60 years	>75 years	3.04	2.51–3.68	<0.001
Age 60–75 years	>75 years	1.68	1.37–2.07	<0.001
High triage priority (levels 1–2)	Low triage priority (levels 3–5)	0.71	0.61–0.82	<0.001
Morning admission (7 a.m.–2 p.m.)	Night admission (9 p.m.–7 a.m.)	0.84	0.72–0.96	0.013
Afternoon admission (2 p.m.–9 p.m.)	Night admission (9 p.m.–7 a.m.)	1.23	1.08–1.41	0.002
Study year: 2020	2019	0.58	0.49–0.69	<0.001
Study year: 2021	2019	0.91	0.81–1.03	0.142
Study setting: Italy	Spain	1.29	1.15–1.45	<0.001

## Data Availability

The raw data supporting the conclusions of this article will be made available by the authors on request.

## Abbreviations

The following abbreviations are used in this manuscript:

ANOVA	Analysis of Variance
aOR	Adjusted Odds Ratio
CI	Confidence Interval
CNS	Central Nervous System
COVID-19	Coronavirus Disease 2019
CV	Cardiovascular
ED	Emergency Department
EMS	Emergency Medical Service
ENT	Ear–Nose–Throat
ESI	Emergency Severity Index
HS	Healthcare System
IBM	International Business Machines
ICD-9-CM	International Classification of Diseases, 9th Revision, Clinical Modification
ICD-10-CM	International Classification of Diseases, 10th Revision, Clinical Modification
LWBS	Left Without Being Seen
OR	Odds Ratio
SD	Standard Deviation
SPSS	Statistical Package for the Social Sciences (SPSS), version 26 (IBM Corp.)
